# Relationship altered between functional T1ρ and BOLD signals in bipolar disorder

**DOI:** 10.1002/brb3.802

**Published:** 2017-09-14

**Authors:** Joseph J. Shaffer, Casey P. Johnson, Jeffrey D. Long, Jess G. Fiedorowicz, Gary E. Christensen, John A. Wemmie, Vincent A. Magnotta

**Affiliations:** ^1^ Department of Radiology University of Iowa Iowa City IA USA; ^2^ Department of Psychiatry University of Iowa Iowa City IA USA; ^3^ Department of Biostatistics University of Iowa Iowa City IA USA; ^4^ Department of Epidemiology University of Iowa Iowa City IA USA; ^5^ Department of Internal Medicine University of Iowa Iowa City IA USA; ^6^ Department of Electrical and Computer Engineering University of Iowa Iowa City IA USA; ^7^ Department of Radiation Oncology University of Iowa Iowa City IA USA; ^8^ Department of Veterans Affairs Medical Center Iowa City IA USA; ^9^ Department of Molecular Physiology and Biophysics University of Iowa Iowa City IA USA; ^10^ Department of Neurosurgery University of Iowa Iowa City IA USA; ^11^ Iowa Neuroscience Institute University of Iowa Iowa City IA USA; ^12^ Department of Biomedical Engineering University of Iowa Iowa City IA USA

**Keywords:** bipolar disorder, BOLD, fMRI, T1ρ

## Abstract

**Introduction:**

Functional neuroimaging typically relies on the blood‐oxygen‐level–dependent (BOLD) contrast, which is sensitive to the influx of oxygenated blood following neuronal activity. A new method, functional T1 relaxation in the rotating frame (fT1ρ) is thought to reflect changes in local brain metabolism, likely pH, and may more directly measure neuronal activity. These two methods were applied to study activation of the visual cortex in participants with bipolar disorder as compared to controls.

**Methods:**

Thirty‐nine participants with bipolar disorder and 32 healthy controls underwent functional neuroimaging during a flashing checkerboard paradigm. Functional images were acquired in alternating blocks of BOLD and fT1ρ. Linear mixed‐effect models were used to examine the relationship between these two functional imaging modalities and to test whether that relationship was altered in bipolar disorder.

**Results:**

BOLD and fT1ρ signal were strongly related in visual and cerebellar areas during the task in controls. The relationship between these two measures was reduced in bipolar disorder within the visual areas, cerebellum, striatum, and thalamus.

**Conclusions:**

These results support a distinct mechanisms underlying BOLD and fT1ρ signals. The weakened relationship between these imaging modalities may provide a novel tool for measuring pathology in bipolar disorder and other psychiatric illnesses.

## 1 INTRODUCTION

Functional magnetic resonance imaging (fMRI) has relied predominantly on the blood‐oxygen‐level–dependent (BOLD) signal to measure changes in brain activation during the completion of behavioral tasks and during resting state. Functional BOLD images are typically acquired using T2*‐weighted imaging, and data analysis involves measuring the fit between the data and an estimated hemodynamic response generated by convolving a canonical hemodynamic response function with the paradigm. However, the BOLD signal is not a direct measure of neuronal activity and is instead dependent on localized changes in blood oxygenation that occurs approximately 4 s after neuronal activation, often detected downstream from the true site of neural activity (Baumann et al., 2010). As a result, the BOLD signal is limited in its temporal and spatial resolution.

An alternative fMRI approach to BOLD was recently proposed based on T1 relaxation in the rotating frame (T1ρ), called *functional T1*ρ *mapping* (fT1ρ) (Jin & Kim, 2013; Johnson, Heo, Thedens, Wemmie, & Magnotta, 2014; Magnotta et al., 2012). This technique aims to quantitatively map the spin‐lock–based T1ρ relaxation time temporally and is sensitive to chemical exchange of protons between water and amide, hydroxyl, and/or amine groups (Jin, Autio, Obata, & Kim, 2011). This chemical exchange is influenced by pH and metabolite concentration (e.g., glucose and glutamate) (Jin et al., 2011; Kettunen, Gröhn, Silvennoinen, Penttonen, & Kauppinen, 2002), which have been shown to change in response to neural activation prior to changes in blood flow (Belanger, Allaman, & Magistretti, 2011). T1ρ relaxation is also sensitive to stimulus‐induced rotary saturation (SIRS), which may directly measure neuronal currents (Witzel, Lin, Rosen, & Wald, 2008). Thus, quantification of T1ρ relaxation temporally is hypothesized to provide a means to detect an early, localized, and nonhemodynamic tissue response to brain activation. In addition to pH, fT1ρ is sensitive to cerebral blood volume, which may contribute a significant hemodynamic signal to the fT1ρ response (Heo, Wemmie, Thedens, & Magnotta, 2014; Hulvershorn et al., 2005; Jin & Kim, 2013; Johnson et al., 2014). However, studies that have masked this hemodynamic contribution to fT1ρ have found that tissue provides the majority of the signal (Heo, Wemmie, Johnson, Thedens, & Magnotta, 2015; Heo et al., 2014; Jin & Kim, 2013). Despite the hemodynamic contribution, fT1ρ mapping provides a potentially complementary method to traditional BOLD fMRI to study brain activation. The potential uniqueness of these two methods have recently been demonstrated in experiments of their timing of functional response (Heo et al., 2015) as well as in a preliminary study in panic disorder (Magnotta, Johnson, Follmer, & Wemmie, 2014).

fT1ρ mapping is of particular interest to study in bipolar disorder because the illness has been associated with metabolic abnormalities, in particular mitochondrial dysfunction (Clay, Sillivan, & Konradi, 2011; Kato, 2007; Stork & Renshaw, 2005). MR spectroscopy studies have shown that baseline pH is reduced (i.e., more acidic) in the anterior cingulate in people with bipolar disorder in the euthymic state compared to normal controls (Kato, Kunugi, Nanko, & Kato, 2000; Kato et al., 1998). Furthermore, a recent study using static, whole‐brain, high‐resolution quantitative T1ρ mapping detected elevated T1ρ relaxation times in the cerebellum and cerebral white matter consistent with reduced basal pH in the euthymic state of people with bipolar disorder compared to healthy controls (Johnson et al., 2015a; Johnson et al., 2015b). These baseline differences in pH and metabolic state may therefore result in differences in functional activity.

It has previously been shown that both BOLD and fT1ρ signals reflect functional activity in vivo (Hulvershorn et al., 2005; Jin & Kim, 2013; Magnotta et al., 2012), however, it remains unclear how these two imaging methods relate to each other (Magnotta et al., 2014). In this exploratory study, we investigated the relationship between BOLD and fT1ρ activation in response to a flashing checkerboard stimulus in participants with bipolar disorder and in matched healthy controls. We expect that differences between these two signals may reflect the distinct sources of fT1ρ and BOLD (i.e., tissue metabolism vs. vascular response). Therefore, we tested whether an altered relationship between these imaging modalities was present in bipolar disorder, which could reflect disease‐related processes.

## 2 METHODS

### 2.1 Participants

A total of 39 participants with bipolar I disorder (23 males; 16 females; mean age 39 ± 14 years) and 32 healthy control participants with no history of psychiatric illness (19 males; 13 females; mean age 42 ± 13 years) underwent functional neuroimaging. Twelve of the participants with bipolar disorder underwent multiple imaging sessions in different mood states (nine participants in two states and three participants in three states), resulting in a total of 54 studies (30 males; 24 females; mean age 41 ± 13 years) consisting of 23, 15, and 16 participants in euthymic, depressed, and manic mood states, respectively. Detailed demographic information is shown in Table 1. Participants were excluded for a history of head trauma, neurological illness, heart disease, or substance abuse as well as contraindications for MRI. Healthy controls were also excluded for a history of psychiatric illness. Clinical diagnosis of bipolar I disorder was confirmed by psychiatric evaluation based on DSM‐IV‐TR criteria (JGF). All participants provided written informed consent according to guidelines established by the University of Iowa Institutional Review Board.

**Table 1:** Demographics of imaging sample

	HC	BD		BD v. HC
# Scans	32	54		
Age				
Mean	42.1	41.1	*t* (*df*)	−0.34(84)
Std. Dev.	12.5	12.5	*p*	.74
Gender				
Male	19	30	χ^2^ (*df*)	0.12(1)
Female	13	24	*p*	.73
Race				
American Indian	0	4	χ^2^ (*df*)	4.14(3)
Asian	0	1	*p*	.25
African American	0	1		
White	32	44		
Ethnicity				
Hispanic	0	1	χ^2^ (*df*)	0.72(1)
Not Hispanic	32	44	*p*	.40
Education				
Mean	16.6	13.8	*t* (*df*)	−5.94(84)
Std. Dev.	2.1	2.1	*p*	<.01*
Handedness				
Right	28	44	χ^2^ (*df*)	1.87(2)
Left	4	7	*p*	.39
Ambidextrous	0	3		
MADRS				
Mean	0.41	11.80		
Std. Dev	0.67	11.78		
YMRS				
Mean	0.06	9.74		
Std. Dev.	0.35	11.71		
Medication class				
Lithium	0	24 (44.4%)	χ^2^ (*df*)	19.73(1)
			*p*	<.01*
Anti‐Convulsants	0	17 (31.5%)	χ^2^ (*df*)	12.56(1)
			*p*	<.01*
Anti‐Depressants	0	23 (42.6%)	χ^2^ (*df*)	18.61(1)
			*p*	<.01*
Anti‐Psychotics	0	24 (44.4%)	χ^2^ (*df*)	19.73(1)
			*p*	<.01*
Sedative‐Hypnotics	0	26 (48.1%)	χ^2^ (*df*)	22.08(1)
			*p*	<.01*

HC, healthy control group; BD, bipolar disorder group; *p < .05.

### 2.2 Flashing checkerboard task

During the flashing checkerboard task, participants were presented with alternating blocks of either a black screen (four blocks) or a black and white checkerboard pattern that “flashed” by inverting the colors at a rate of 4 Hz (three blocks). Each block lasted for 40 s and a total of seven blocks were presented during each run of the flashing checkerboard task. To confirm that participants were attending to the stimuli, a red square was presented every 4 s during the flashing checkerboard blocks, to which the participants responded by pressing a button. Participants completed a total of five identical runs of the task, which alternated between fT1ρ (three runs) and BOLD (two runs) sequences.

### 2.3 Image acquisition

All participants were imaged using a 3T MRI system (Magnetom Tim Trio; Siemens Healthcare; Erlangen, Germany) with a vendor‐provided 12‐channel receiver head coil. First, high‐resolution T1‐ and T2‐weighted anatomical images were acquired to align the participants’ fT1ρ and BOLD data to a common atlas space for voxel‐wise comparison. The T1‐weighted sequence parameters were as follows: coronal 3D MP‐RAGE; field of view = 256 mm^3^; sampling matrix = 256 × 256 × 256; resolution = 1.0 mm^3^; TR = 2,530 ms; TE = 2.8 ms; TI = 909 ms; flip angle = 10°; BW = 180 Hz/px; and R = 2 GRAPPA. The T2‐weighted sequence parameters were as follows: sagittal 3D SPACE; field of view = 260 × 228 × 176 mm^3^; sampling matrix = 256 × 230 × 176; resolution = 1.0 mm^3^; TR = 4,000 ms; TE = 406 ms; BW = 592 Hz/px; turbo factor = 121; slice turbo factor = 2; and R = 2 GRAPPA.

Next, fT1ρ and BOLD time series were acquired in conjunction with the flashing checkerboard stimulus. The fT1ρ sequence used a previously described technique to acquire quantitative T1ρ relaxation maps very rapidly using a spin‐echo echo‐planar imaging (SE‐EPI) sequence with a very short time interval between spin‐lock preparation blocks (Johnson et al., 2014). In this study, two spin‐lock‐weighted images at 10 imaging slices were acquired every 4.0 s. Spin‐lock times (TSLs) were 10 and 50 ms and spin‐lock amplitude (γB_1_/2π) was 213 Hz. Other sequence parameters were as follows: field of view = 240 × 240 mm^2^; sampling matrix = 64 × 64 (single shot); slice thickness/gap = 5.0/1.25 mm; TR = 2,000 ms; TE = 15 ms; BW = 1,954 Hz/px; partial Fourier = 5/8; fat saturation; and 140 measurements. The imaging slices were acquired in an axial‐oblique orientation and angled such that the most inferior slice was positioned just below the base of the frontal and occipital lobes (Fig. S1). The BOLD sequence used a standard T2*‐weighted gradient‐echo echo‐planar imaging (GRE‐EPI) imaging sequence with parameters: field of view = 220 × 220 mm^2^; sampling matrix = 64 × 64 (single shot); 30 slices; slice thickness/gap = 4.0/1.0 mm; TR = 2,000 ms; TE = 30 ms; BW = 2,004 Hz/px; fat saturation; and 140 measurements. The slices were oriented to match that of the fT1ρ acquisition.

### 2.4 Image analysis

For each participant, fT1ρ and BOLD time series were processed using Analysis of Functional NeuroImages (AFNI) (Cox, 1996). Images from the three fT1ρ runs were registered, skull stripped, and spatially smoothed with a 5.0 mm FWHM Gaussian filter. Prior to smoothing, the first two of the 10 acquired slices were removed to reduce the influence of steady‐state effects (Johnson et al., 2014). Next, the time series of T1ρ maps was calculated by fitting the 10 ms and temporally interpolated 50 ms spin‐lock time images to a mono‐exponential signal decay model, as previously described (Johnson et al., 2014). The average percent change in T1ρ values at each voxel in response to the flashing checkerboard stimulus was then calculated using a general linear model with the timing of the task's block paradigm as the assumed response profile, second‐order baseline correction, and regression of motion nuisance parameters. Images from the two BOLD runs were de‐spiked and similarly registered and spatially smoothed. The same general linear model was used as for fT1ρ to calculate the average percent change in BOLD signal activation at each voxel during the flashing checkerboard stimulus.

Anatomical T1‐ and T2‐weighted images from each participant were processed using BRAINS AutoWorkup (Pierson et al., 2011) and Advanced Normalization Tools (ANTS) to generate a deformable transformation to a common atlas space. This transform was applied to register each functional run to the common atlas space (Halle et al., 2015), resulting in voxel‐wise alignment of all participants’ data. To reduce the influence of spatial variability in the placement of functional imaging slices between participants, all voxels that did not have at least 95% overlap of both fT1ρ and BOLD data from all participants were masked and removed from subsequent analysis. Additionally, voxels were masked to only include brain tissue voxels as defined by the common atlas (Halle et al., 2015). Images were transformed to the Montreal Neurological Imaging (MNI) atlas space (Evans et al., 1993) after analyses were completed to provide a standard coordinate system for publication.

### 2.5 Statistical analysis

The relationship among BOLD activation, fT1ρ activation, and group (bipolar disorder vs. healthy control) was measured for each voxel using multiple linear regression. As noted, 12 of the same participants were imaged in different mood states resulting in the nesting of scans within persons. To account for repeated measurements, linear mixed model (LMM) regression was performed using the *fitlme* function in MATLAB (R2015a; Mathworks; Natick, Massachussetts). Two separate models were tested. In the null model (Equation (1)), fT1ρ and group (0 =  healthy control, 1 =  bipolar) were used as fixed variables to predict BOLD, while participant age and gender were included as covariates (Equation (1)). The experimental model (Equation (2)) was identical to the null model with the addition of an interaction between Group and fT1ρ. An individual‐specific random effect (intercept only) was included to account for the dependency due to nesting. Suppose that $BOLD_{ij}$ is the BOLD signal for the $i^{th}$ individual (i=1,…,39) and the $j^{th}$ measurement $\left(j=1,\ldots ,T_{i}\right)$ . The LMM models were as follows:

(1) $$BOLD_{ij}=\alpha_{i}+\alpha +\beta \cdot Group_{i}+\gamma \cdot fT1\rho_{i}+\delta \cdot Age_{i}+\pi \cdot Sex_{i}+e_{ij},$$

(2) $$BOLD_{ij}=\alpha_{i}+\alpha +\beta \cdot Group_{i}+\gamma \cdot fT1\rho_{i}+\delta \cdot Age_{i}+\pi \cdot Sex_{i}+\psi \cdot \left(Group_{i}\cdot fT1\rho_{i}\right)+e_{ij}.$$

The Greek letters are fixed effects, withψ representing the Group by fT1ρ interaction of interest; $a_{i}$ is the individual‐specific random effect, which is assumed to have a zero‐mean normal distribution; and *e* is random error, assumed to have a zero‐mean normal distribution and constant over the repeated measurements (where applicable). Parameters were estimated using maximum likelihood methods (Verbeke & Molenberghs, 2000), and the likelihood ratio test (LRT) was used to test the null hypothesis of no group × fT1ρ difference (i.e., $H_{0}:\psi =0$ ). Only voxels where the LRT was significant (*p *<* *.05) were included in the results. This mask is shown in Fig. S2. We then extracted the *t*‐statistic for the group × fT1ρ interaction from the experimental model (ψ from Equation (2)). A cluster‐based approach was used for correcting for multiple comparisons. The *3dClustSim* function from AFNI (version AFNI_2011_12_21_1014, compiled Sept., 2015) was used to simulate the necessary number of contiguous significant voxels needed to maintain alpha = 0.05. The required cluster size was calculated to be 1.44 cm^3^.

### 2.6 Post hoc analysis

In order to help us interpret the results of the LMM analysis, we performed a post hoc comparison of mean activations within each cluster that was significantly related to the interaction of group and fT1ρ effects on the BOLD signal. BOLD and fT1ρ images were normalized for each participant by converting percent signal change into a z‐statistic. Mean activation was then calculated in each cluster for each participant. Means were compared between groups (Bipolar, Healthy Control) within each imaging modality (BOLD, fT1ρ), between groups across imaging modalities, between imaging modalities within groups, and between imaging modalities across groups using a two‐sample *t*‐test.

## 3 RESULTS

### 3.1 Effect of bipolar disorder on the relationship between BOLD and fT1ρ

**Figure 1:**
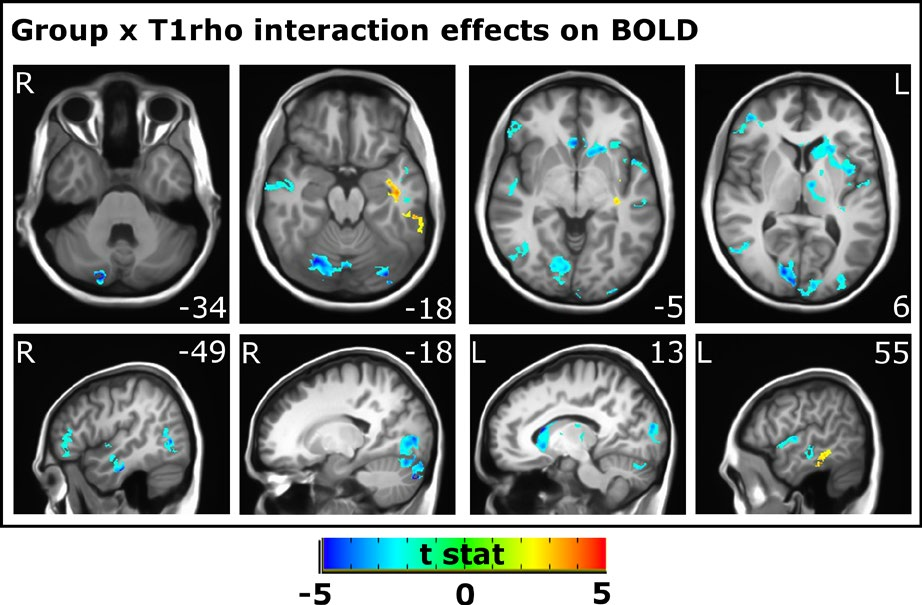
Clusters where there was a significant effect of the interaction between Group and T1ρ on BOLD signal. Clusters where the relationship between T1ρ and BOLD were reduced in bipolar disorder are shown in blue and clusters where the relationship between T1ρ and BOLD was increased in bipolar disorder are shown in red/yellow. MNI coordinates are provided for the Z plane in axial images and X plane in sagittal images. Left (L) and right (R) are indicated as well

A weakened relationship (i.e., decoupling) between the fT1ρ signal and BOLD signal was present in bipolar disorder in several regions (Table 2, Figure 1) including the left caudate, left thalamus, bilateral visual cortex, left occipital pole, right lateral occipital cortex, bilateral cerebellum, right inferior temporal gyrus, and left middle and superior temporal gyri (note that a negative *t*‐value reflects BOLD‐fT1ρ decoupling in the bipolar group due to the way that the groups were entered into the LMM model, with bipolar group = 1 and healthy control group = 0). Conversely, coupling between BOLD and fT1ρ was enhanced in bipolar disorder in the left inferior and middle temporal gyri.

**Table 2:** Spatial locations of significant clusters

Cluster	Size (mm^3^)	Cluster		Peak				Regions
		Mean *t*	*SD*	X	Y	Z	*t*	
fT1ρ								
1	7,140	3.64	0.91	−17	86	−35	8.81	R visual areas & R cerebellum
2	3,344	3.66	0.74	10	96	4	6.48	L visual areas
3	1,572	4.23	1.12	33	85	−17	8.53	L Cerebellum
Group × fT1ρ								
1	18,837	−2.94	0.61	11	−17	13	−7.10	L Caudate, L Thalamus
2	7,127	−3.18	0.72	−17	86	−35	−9.55	R Cerebellum & R Visual Areas
3	3,288	−2.99	0.56	19	92	13	−5.45	L Visual Areas
4	2,670	3.01	0.56	42	16	−19	5.32	L mid & L inf Temporal Gyri
5	2,616	−2.67	0.35	53	8	4	−4.20	L sup & mid Temporal Gyri
6	2,364	−2.77	0.42	−45	−46	4	−4.69	R inf & mid frontal gyrus
7	1,696	−2.87	0.60	−48	17	−27	−5.92	R inf Temporal Gyrus
8	1,623	−2.86	0.49	−49	67	1	−5.21	R lateral Occipital
9	1,533	−3.17	0.71	33	85	−17	−6.04	L Cerebellum & L Occipital Pole

R, right; L, left; inf, inferior; mid, middle; sup, superior.

### 3.2 Post hoc analysis

In order to rule out the possibility that the observed decoupling between BOLD and fT1ρ in bipolar disorder was due to a failure of one imaging modality to identify group differences that were present in the other imaging modality, we performed a series of post hoc tests where we compared the normalized means of functional activity within the nine clusters identified by the LMM analysis. The anatomical locations of the clusters are described in Table 2, and the results of the contrasts are presented in Table 3.

**Table 3:** Post hoc tests of clusters identified by regression model

	BOLD				T1ρ				BOLD		T1ρ		Both		BD		HC		Both	
	BD		HC		BD		HC		BD v HC		BD v HC		BD v HC		BOLD v T1ρ		BOLD v T1ρ		BOLD v T1ρ	
	*M*	*SD*	*M*	*SD*	*M*	*SD*	*M*	*SD*	*t*	*p*	*t*	*p*	*t*	*p*	*t*	*p*	*t*	*p*	*t*	*p*
1	−0.10	0.21	−0.10	0.14	−0.05	0.15	−0.04	0.12	−0.11	.92	−0.16	.87	−0.18	.86	−1.58	.12	−1.75	.09	−2.23	.03*
2	2.16	0.73	2.22	0.91	0.16	0.20	0.12	0.27	−0.35	.73	0.93	.36	−0.04	.97	19.41	.00**	12.58	.00**	22.83	.00**
3	1.51	0.79	1.81	0.78	0.12	0.23	0.16	0.17	−1.71	.09	−0.85	.40	−1.14	.26	12.37	.00**	11.77	.00**	16.77	.00**
4	−0.19	0.18	−0.24	0.11	−0.03	0.15	−0.05	0.17	1.29	.20	0.55	.58	1.15	.25	−4.99	.00**	−5.23	.00**	−7.04	.00**
5	−0.15	0.32	−0.32	0.24	0.00	0.35	−0.07	0.22	2.66	.01**	0.97	.33	2.42	.02*	−2.28	.03*	−4.39	.00**	−3.99	.00**
6	0.02	0.38	0.00	0.31	0.01	0.25	0.03	0.10	0.20	.85	−0.43	.67	−0.04	.97	0.04	.97	−0.57	.57	−0.24	.81
7	−0.39	0.25	−0.41	0.19	−0.04	0.26	−0.10	0.17	0.48	.64	1.20	.23	0.96	.34	−7.17	.00**	−6.98	.00**	−9.63	.00**
8	0.00	0.53	0.12	0.54	0.05	0.14	0.03	0.22	−0.97	.33	0.48	.63	−0.78	.44	−0.58	.57	0.89	.38	0.11	.91
9	2.09	1.16	2.12	1.27	0.26	0.50	0.26	0.32	−0.09	.93	−0.03	.98	−0.07	.95	10.67	.00**	8.03	.00**	13.42	.00**

*p < .05, **p < .01; BD, bipolar disorder group; HC, healthy control group; *M*, mean; *SD*, standard deviation.

When we compared the bipolar and healthy control groups within each imaging modality, we found no group differences in fT1ρ and only found group differences in the BOLD data for Cluster 5 (left superior and middle temporal gyri). When we combined the BOLD and fT1ρ data, we also found that activity in Cluster 5 was significantly increased in bipolar disorder. These findings suggest that neither BOLD nor fT1ρ alone is particularly sensitive to group differences within these regions.

When we compared the BOLD and fT1ρ data within each group and combined across all groups, we found that significant differences were present in Clusters 2, 3, 4, 5, 7, and 9 in the bipolar group, healthy control group, and when both groups were combined. When both groups were combined, significant differences between BOLD and T1ρ were also present in Cluster 1. These comparisons suggest that decoupling between the BOLD and fT1ρ responses is likely to underlie the results of the LMM analysis.

## 4 DISCUSSION

We compared the relationship between fT1ρ and BOLD imaging between participants with bipolar disorder and healthy controls. We found that the relationship between these two imaging modalities was altered in bipolar disorder, suggesting that differences between fT1ρ and BOLD signals may reflect the underlying pathophysiology of the illness.

The physiological source of BOLD signal has been well‐characterized by previous research and it is known to reflect changes in blood oxygenation that occur following coherent neuronal activity (Baumann et al., 2010; Logothetis, Pauls, Augath, Trinath, & Oeltermann, 2001; Magri, Schridde, Murayama, Panzeri, & Logothetis, 2012). However, a major limitation of BOLD imaging is that changes in blood flow are not a direct measurement of neural activity, likely requiring the involvement of astrocyte‐mediated signaling pathways (Rossi, 2006; Takano et al., 2006) and occurring approximately 4–6 s after neuronal activity (Baumann et al., 2010). In contrast, the physiological source of T1ρ is less well‐understood. Prior studies have shown that T1ρ is sensitive to changes in pH, with signal increasing with acidity (Heo et al., 2014; Kettunen et al., 2002). Because of this, fT1ρ signal may reflect increases in acidic metabolites such as H^+^, glutamate, and lactate in tissue following neuronal activation (Belanger et al., 2011). We would then expect that neuronal activation would result in an increase in both BOLD and fT1ρ signal.

However, when we explored the interactions between group and imaging modality, we found that the relationship between fT1ρ and BOLD differed between participants with bipolar disorder and healthy controls in a number of brain regions. For the most part, these differences took the form of a weaker relationship between fT1ρ and BOLD in the bipolar group versus the healthy control group (i.e., the imaging modalities were decoupled). These regions included the visual cortex, cerebellum, striatum, thalamus, medial prefrontal, and temporal cortex regions. However, there were also two regions that had a stronger relationship between these imaging modalities in the bipolar versus healthy control group (i.e., coupling was enhanced), the left temporal pole and left inferior temporal gyrus. Many of these regions have been previously implicated by functional imaging studies as having a role in bipolar disorder (Cerullo, Adler, Delbello, & Strakowski, 2009; Gruber, Rogowska, & Yurgelun‐Todd, 2004; Keener & Phillips, 2007; Maletic & Raison, 2014; Strakowski et al., 2011; Townsend et al., 2012; Whitton, Treadway, & Pizzagalli, 2015; Yoshimura et al., 2014), which suggests that the altered relationship between fT1ρ and BOLD is related to the illness. For instance, numerous studies have shown that functional activity in the striatum is altered in bipolar disorder during the completion of reward tasks (Caseras, Lawrence, Murphy, Wise, & Phillips, 2013; Whitton et al., 2015; Yip, Worhunsky, Rogers, & Goodwin, 2014) and reduced during fear perception tasks (Killgore, Gruber, & Yurgelun‐Todd, 2008). Similarly, functional activity in the anterior cingulate was also reduced during the same fear perception task (Killgore et al., 2008) and during attention tasks (Gruber et al., 2004). Likewise, reduced thalamic volume (Radenbach et al., 2010) and reduced connectivity between the thalamus and striatum (Teng et al., 2014) have been identified in bipolar disorder. However, the results of our post hoc analysis show that neither BOLD nor fT1ρ showed group differences within these regions, suggesting that our findings are primarily driven by differences between the imaging modalities (i.e., decoupling between BOLD and fT1ρ) rather than group differences being present in one imaging modality and absent in the other (i.e., a failure of one method to find group differences). The absence of group differences in these areas may be explained by the fact that the flashing checkerboard task does not directly interrogate these networks, however, the altered coupling between BOLD and fT1ρ suggests that dysfunction may be present in these regions even when they are not specifically activated by a task. For example, through the accumulation of metabolites that fT1ρ is sensitive to, but that are relatively independent from neuronal activation in bipolar disorder, which may be related to group differences in quantitative T1ρ found in previous work by our group in this population (Johnson et al., 2015a).

The mechanism for decoupling between these imaging modalities, and furthermore, how it relates to bipolar disorder, is currently unclear. One possibility is that this altered coupling occurs because fT1ρ and BOLD signal vary in terms of either their temporal or spatial distribution. However, in this study, both the temporal (40s blocks) and spatial resolution (>2.2 cm^3^ of tissue) of our significant findings are quite coarse and are likely greater than any expected variations differences between the two imaging modalities.

Another possibility is that disease‐related changes in the relationship between fT1ρ and BOLD occur due to altered signaling pathways. The BOLD signal relies on the recruitment of blood flow following neuronal activation. This recruitment involves a multi‐step signaling pathway that is dependent on astrocyte activity. If, as we expect, fT1ρ reflects changes in acidic metabolites (and therefore pH) in tissue that occur directly as a result of neuronal activation while BOLD relies on a less direct signaling pathway, then a disease‐related disruption in this pathway may explain the decoupling that we are seeing between the two imaging modalities in bipolar disorder.

A third possibility is that this decoupling is due to metabolic abnormalities. Numerous studies have identified disruptions in metabolic pathways in participants with bipolar disorder compared to healthy controls (Cecil, DelBello, Morey, & Strakowski, 2002; Dusi, Cecchetto, & Brambilla, 2016; Sikoglu et al., 2013; Stork & Renshaw, 2005; Yuksel et al., 2015). These disruptions seem to be focused on mitochondrial mechanisms for generating ATP including oxidative metabolism (Dusi et al., 2016; Stork & Renshaw, 2005) and the use of phosphocreatine as an energy source (Sikoglu et al., 2013; Yuksel et al., 2015). Collectively, these deficits suggest that in bipolar disorder, neurons are more heavily reliant on glycolysis for the creation of ATP (Stork & Renshaw, 2005). Likewise, PET studies have shown that flashing checkerboard stimuli result in an increase in glucose usage, but not oxygen usage in visual cortex (Belanger et al., 2011). This increased reliance on glycolysis for energy may result in increased oxidative stress (Belanger et al., 2011; Dusi et al., 2016), which is known to occur in bipolar disorder (Brown, Andreazza, & Young, 2014; Salim, 2014; Tang & Wang, 2012), and may result in a number of other brain changes including changes in neuroplasticity and functional activation (Berk et al., 2011; Tang & Wang, 2012). Such abnormalities in glucose consumption, metabolism, and oxidative stress in bipolar patients might differentially affect the mechanisms underlying fT1ρ and BOLD responses. In order to pinpoint the cause(s) of the abnormal relationship between fT1ρ and BOLD in bipolar disorder patients, more work is needed to better understand the fT1ρ response and the differences between fT1ρ and BOLD.

### 4.1 Limitations

This study was carried out using participants in several mood states and on different medications. Each of these subgroups may have a unique response and it would be interesting to study each of these mood states separately in a larger study.

Because of acquisition time constraints, there was limited brain coverage of fT1ρ mapping. This also required the use of a block‐based design, whereas an event‐based design would provide better temporal resolution for comparing the BOLD and fT1ρ signal during the flashing checkerboard. Other limitations of this sequence and potential options to improve imaging efficiency are discussed in detail in Johnson et al. (Johnson et al., 2014). It would also be interesting to compare eccentricity mapping between fT1ρ mapping and BOLD as previously studied in healthy participants using data from participants with bipolar disorder (Heo et al., 2015).

## 5 CONCLUSION

We explored the relationship between a novel functional imaging technique, T1ρ, and the more traditional T2*‐based BOLD imaging response during a flashing checkerboard paradigm and compared that relationship between participants with bipolar disorder and healthy controls. Overall, our results support the use of T1ρ as a functional neuroimaging method for use in clinical populations, as there was a strong positive relationship between fT1ρ and BOLD activity in visual areas thought to be involved in the flashing checkerboard paradigm. Importantly, we identified a decoupling of functional BOLD and T1ρ signal in participants with bipolar disorder, which may reflect a differential sensitivity between these imaging modalities to the pathophysiology of bipolar disorder. These findings suggest that fT1ρ may provide a unique tool for measuring other aspects of functional activity; and, when used in conjunction with BOLD imaging, fT1ρ may help to elucidate disease mechanisms that are not reflected in BOLD signal alone.

## CONFLICT OF INTEREST

The authors do not have any conflicts of interest to disclose.
