# Supplementary figures and images for: Relationship altered between functional T1ρ and BOLD signals in bipolar disorder
Source: Brain Behav. 2017 Sep 14;7(10):e00802. doi: 10.1002/brb3.802 (PMC5651386; doi:10.1002/brb3.802)

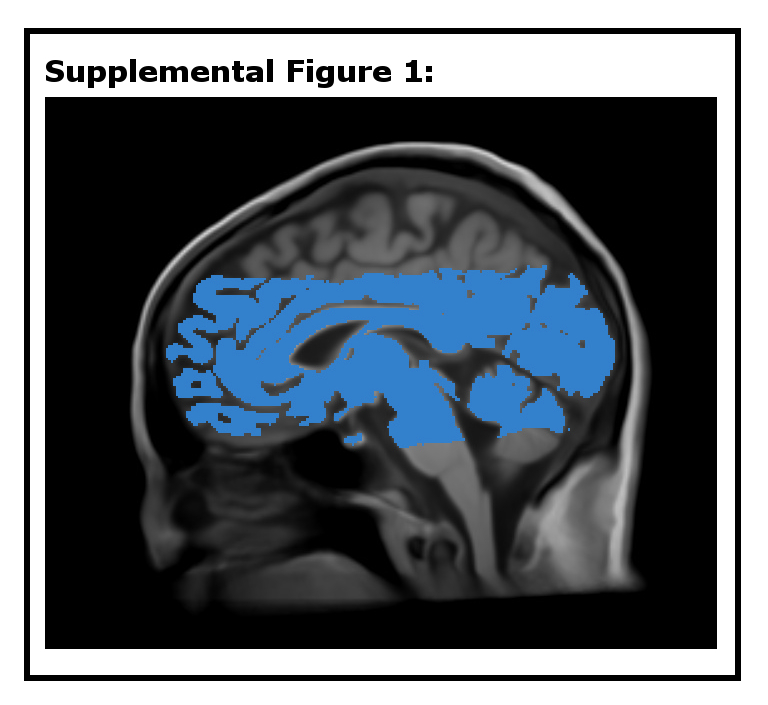

Supplement: Supplementary file 1 [file BRB3-7-e00802-s001.jpg]

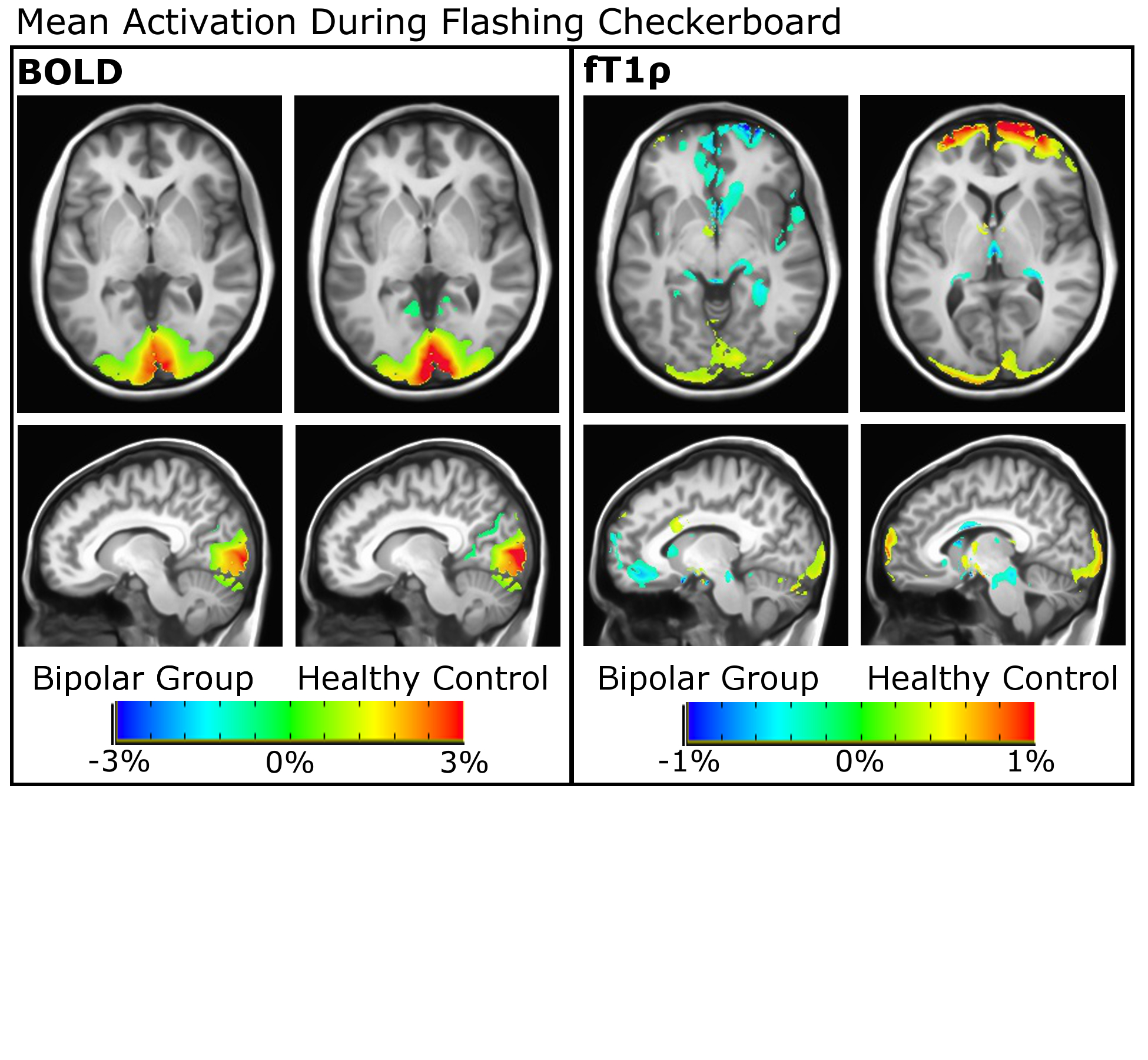

Supplement: Supplementary file 2 [file BRB3-7-e00802-s002.jpg]
