# Supplementary material for: Relationship altered between functional T1ρ and BOLD signals in bipolar disorder
Source: Brain Behav. 2017 Sep 14;7(10):e00802. doi: 10.1002/brb3.802 (PMC5651386; doi:10.1002/brb3.802)
Supplement: Supplementary file 3 [file BRB3-7-e00802-s003.docx]

Supp. Fig 1:

Supplemental Figure 1: Mask showing superior-inferior acquisition extent for fT1ρ images. Analyses were restricted to only voxels included in the fT1ρ acquisition.

Supplemental Figure 2: Mean percent signal change map for each group and imaging method. The top 5% of voxels are shown.
